# Impact of background music on the performance of laparoscopy teams

**DOI:** 10.1186/s12909-022-03503-7

**Published:** 2022-06-07

**Authors:** Ying Han, Bin Zheng, Linyong Zhao, Jiankun Hu, Chao Zhang, Ran Xiao, Chunyan Wang, Dan Pu

**Affiliations:** 1 West China Medical Simulation Center, West China Hospital, Sichuan University, Chengdu, Sichuan Province 610041 People’s Republic of China; 2 Department of Surgery, University of Alberta, Alberta, Canada; 3 Department of Gastrointestinal Surgery, West China Hospital, Sichuan University, Chengdu, Sichuan Province People’s Republic of China 610041; 4 West China Academic Exchange Center of Health and Medicine, Chengdu, Sichuan Province People’s Republic of China 610041

**Keywords:** Team performance, Laparoscopic surgery, Acoustic distraction, Video analysis, Simulation, Surgical training

## Abstract

**Background:**

Acoustic conditions in the operating room have different impacts on surgeon’s performance. Their effects on the performance of surgical teams are not well documented. We investigated if laparoscopic teams operating under pleasant acoustic conditions would perform better than under noisy conditions.

**Methods:**

We recruited 114 surgical residents and built 57 two-person teams. Each team was required to perform two laparoscopic tasks (object transportation and collaborative suturing) on a simulation training box under music, neutral, and noisy acoustic conditions. Data were extracted from video recordings of each performance for analysis. Task performance was measured by the duration of time to complete a task and the total number of errors, and objective performance scores. The measures were compared over the three acoustic conditions.

**Results:**

A music environment elicited higher performance scores than a noisy environment for both the object transportation (performance score: 66.3 ± 8.6 vs. 57.6 ± 11.2; *p* < 0.001) and collaborative suturing tasks (78.6 ± 5.4 vs. 67.2 ± 11.1; *p* < 0.001). Task times in the music and noisy environments was subtracted to produce a music-noisy difference time. *Pearson* correlation coefficient analysis showed a significant negative relationship between the team experience score and the music-noisy difference time on the object transportation (*r* = − 0.246, *p* = 0.046) and collaborative suturing tasks (*r* = − 0.248, *p* = 0.044).

**Conclusions:**

As to individuals, music enhances the performance of a laparoscopy team while a noisy environment worsens performance. The negative correlation between team experience and music-noisy difference time suggests that laparoscopy teams composed of experienced surgeons are less likely affected by an acoustic distraction than the noisy teams. Team resistance to acoustic distraction may lead to a new way for assessing team skills.

## Introduction

Music is commonly allowed during surgery in many operating theatres worldwide. Surgical staff has reported that music can reduce stress and increase efficiency [1–4]. Several studies have examined the impact of background music on surgical performance [5–7]. One study showed that when surgeons were allowed to select and play recordings of their preferred music, their stress was reduced and their task performance improved compared with when they had to listen to music selected by others [8]. Another study found that music composed by Mozart evoked a relaxed mood and resulted in improved surgical performance [9] and spatial orientation ability [10].

While some claimed an improvement by playing the preferred music, others denied a positive impact or even reported a negative impact on surgical performance [5, 11, 12]. The definition of preferred music varied between different surgeons. Mozart’s melody was too soporific for some, whereas Michael Jackson’s music was too loud and annoying to others [11–13].

Surgical dexterity deteriorated in a noisy environment [14, 15]. Negative emotions triggered by a noisy environment has led to a deterioration in the cognitive ability of surgeons due to deteriorated working memory and to interference in the decision-making process [16, 17]. People in noisy environments reported difficulty in maintaining focus, thus showing reduced ability in assessing a situation and a tendency for selecting high-risk strategies for resolving a problem without being fully aware of the consequences [8, 18, 19].

The impact of music on an individual is intriguing. However, the impact of music on the performance of a surgical team is complex and remains unclear [20]. Surgery is generally known as a team practice involving surgeons and other healthcare providers [21]. In addition to personal skills, the outcome of any surgical operation largely depends on the quality of team communication and collaboration [22]. In team settings, the positive impact of music may be canceled out by personal choices on their preferred music [23]. On the other hand, negative emotions triggered by unpleasant music can be ‘contagious’ and affect many team members, often resulting in a negative performance by the entire team [16, 24].

The above statement regarding team performance in the operating room is particularly true in the laparoscopic surgical procedure. Unlike open surgical procedure, the laparoscopic surgery required surgeons to perform an operation using long-shaft surgical instruments that insert into the abdominal cavity; the surgical site is captured by a special digital video camera (laparoscope) and displayed to a high-definition monitor. In any laparoscopic procedure, the primary surgeon’s vision is controlled by an assistant surgeon who manipulates and directes the scope. The movement and cognitive synchronization between the primary and the assistant surgeons in laparoscopic surgery is important for the team performance. it also provides an appropriate situation for us to examine the impact of music in surgical team performance.

We report here the findings of our recent study to investigate the impact of different acoustic conditions on the performance of laparoscopic teams. We asked teams of two surgeons to perform two tasks during a simulated laparoscopic under three different background acoustic conditions: pleasant and smooth music, neutral (quiet), and noisy conditions. We hypothesized that 2-surgeon teams working under the smooth music condition would perform better than teams operating in a noisy or neutral environment. The effects would be more noticeable for teams of inexperienced surgeons than for teams of experienced surgeons since experienced surgeons might be less sensitive to an acoustic distraction as inexperienced surgeons.

## Methods

### Study environment

The study was designed and conducted in accordance with the Declaration of Helsinki and competent laws and regulations in China. This controlled laboratory study was conducted at the Medical Simulation Center of West China Hospital of Sichuan University. The study protocol was approved by the University of West China Hospital of Sichuan University Research Ethics Board (2019 Approval No. 1071). Informed consent was obtained from all participants prior to data collection. Each participant provided written consent before entering the study.

### Apparatus

**Fig. 1:**
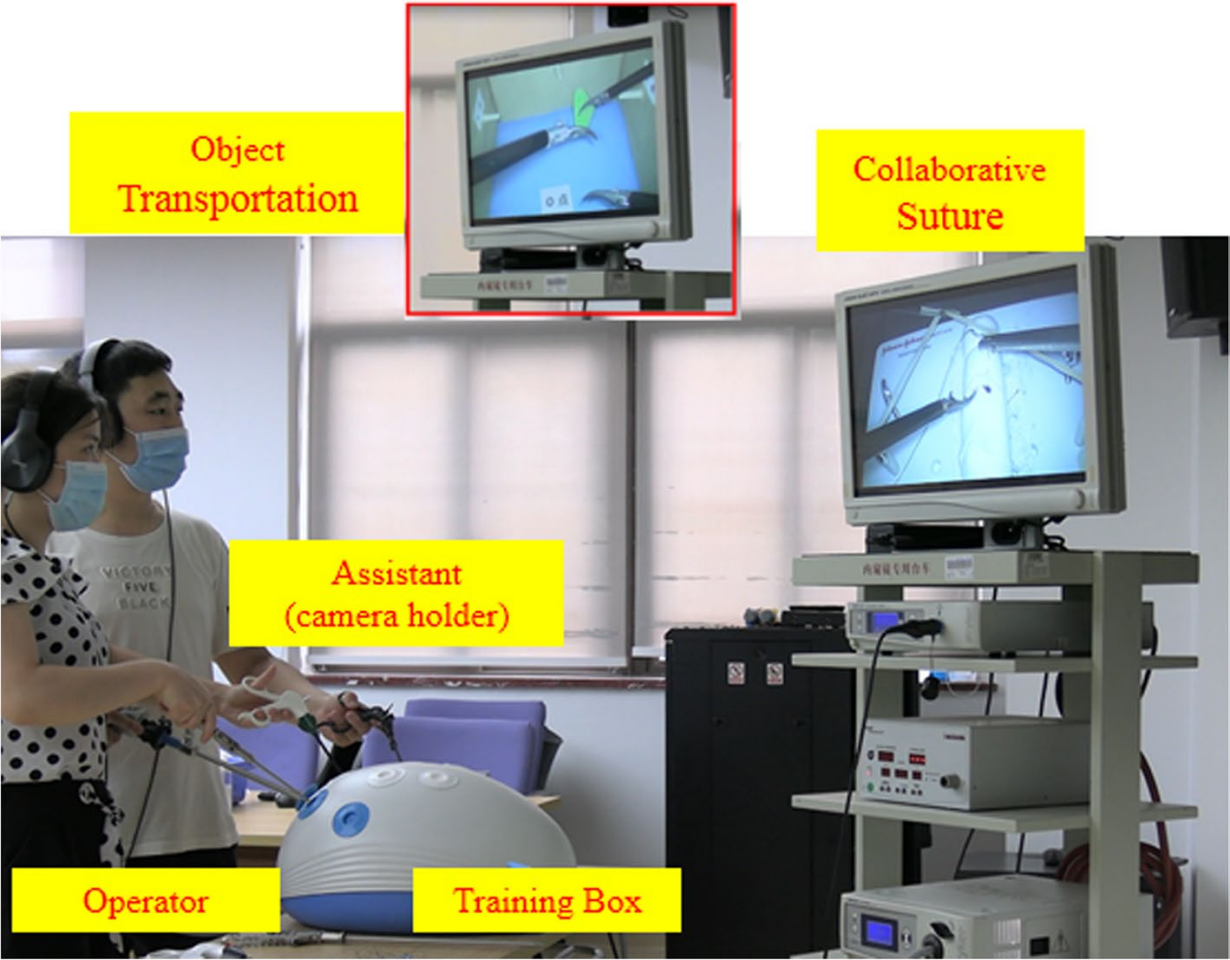
Experimental setting. Two surgeons were assigned to 2-person teams to perform simulated tasks as follows: the object transportation (top panel) and collaborative suturing tasks (right screen). Please note that participants were wearing headsets that delivered three different acoustic conditions

The team performance was done on a laparoscopic training box (SL-PE480, Shinno-Med Inc., Shanghai, China Fig. 1). Three 5 mm diameter endoscopic instruments were inserted through 3 ports to the surgical site. The surgical site was illuminated by a Stryker X8000 light source, captured by a 30-degree laparoscope, and displayed on a 26-in. high-definition monitor (CANON Legria H50FG, Tokyo, Japan). On the object transportation task, the operators used two 5 mm curved graspers (Ethicon Endo-Surgery Inc., Cincinnati, Ohio, USA). On the suturing task, the operator used a pair of needle drivers (ET705R, Ethicon Endo-Surgery Inc., Cincinnati, Ohio, USA) to perform the suturing and was assisted by the assistant who used a 5 mm curved grasper (Ethicon Endo-Surgery Inc., Cincinnati, Ohio, USA).

### Participants

A total of 114 surgical residents who had no experience of simulated laparoscopic training in teams (88 men and 26 women aged 34.3 ± 4.8 years) were recruited from the Department of Surgery Residency Program at the West China Hospital of Sichuan University between May 2020 and October 2020. The hospital is a leading tertiary hospital in China with specialties in minimally invasive surgery. Most participants had at least 4 h of individual laparoscopic training experience with a bench-top laparoscopic training box. Some of the participants had experience performing a complete laparoscopic procedure on a virtual model. The participants were randomized into 57 two-person teams. Table 1 shows the results of a custom-designed survey of the pre-training surgical clinical experience of participants [25]. We also assessed the participants’ moods before operation with the Profile of Mood States (POMS) [26] and the Positive and Negative Affect Schedule (PANAS-SF) questionnaires [27]. The assessment on the mood of surgeons was essential because the impact of music on a human’s performance was mainly mediated by change of mood of a human operator. The POMS and PANAS assessment at the beginning of the experiment helped us to make correct interpretation on the results.

**Table 1:** Task measures, subtask times, and errors that were used for the calculation of total task scores. A list of time and error measures taken from two tasks and the description

	Object Transportation		Collaborative Suture	
	Measure	Description	Measure	Description
**Time**	Total time	Object on to Peg C - Trial start	Total time	Suture cut - Trial start
	Time on peg A	Object on to Peg A - Trial start	Time on preparation	Needle first puncture - Trial start
	Time on peg B	Object on to Peg B - Object on to Peg A	Time on suturing	Beginning of 1st knot tying - Needle first puncture
	Time on peg C	Object on to Peg C - Object on to Peg B	Time on knot 1	Beginning of 2nd knot tying - Beginning of 1st knot tying
			Time on knot 2	Scissor in view - Beginning of 2nd knot tying
			Time on cutting	Thread cut - Scissor in view
**Errors**	# of object drop (+ 3 s)	number of plastic triangle drop during entire trial	# of needle adjustment	Number of needle being picked up, orientation adjustment
	# of object transfer between hands (+ 3 s)	Number of object being transfer between hands of the chief operator	# of needle insert/exiting	Number of attempts of needle inserting and exiting the suture sides
	# of incorrect view (+ 3 s)	number of object or tips of instrument out of the scope view	# of cutting	Number of attempts of cutting thread after knot typing
	# of horizontal line twist (+ 3 s)	Number of times when the scope view is not horizontal.	# of incorrect view	number of object or tips of instrument out of the scope view
	# of instruments collision (+ 3 s)	Number of collision between scope and instruments	# of horizontal line twist	Number of times when the scope view is not horizontal.
	Communication	Silent team (+ 10s), insufficient communication (+ 5 s)	# of instruments collision	Number of collision between scope and instruments
			Communication	Silent team (+10s), insufficient communication (+ 5 s)
			Quality of knot tying	Loose knot (+ 10 s); unsecured knot (+ 5 s)

The study sample size (number of teams) was determined according to a similar investigation. In 2020, Yang et al. reported the effect of different emotions on laparoscopic performance [16]. They asked surgeons-in-training to perform simulated surgical tasks on the Lap Mentor (Simbionix, Tel Aviv, Israel) immediately after watching three movies that evoked different emotions. Surgeons with positive emotions performed a task within a significantly shorter time (13.7 ± 2.5 minutes) than those with negative (18.5 ± 3.8 minutes) and neutral emotions (17.7 ± 3.9 minutes), for approximately 35% differences between the positive-and-negative-emotion groups. We anticipated a modest 20% improvement in our study, since the impact on the performance of a surgical team might not be as strong as the impact on an individual of a team. Using a one-way analysis of variance (ANOVA) model with a two-tailed alpha of 0.05 and a beta of 0.10 (power of 90%), we calculated a minimum of 18 teams needed in each of acoustic conditions to demonstrate significance.

### Team tasks

After the surveys, two random participants arrived at the simulation lab as a team (operator and assistant) and were asked to perform two laparoscopic tasks, object transportation and collaborative suturing. For the object transportation task, the assistant was required to move the 30-degree laparoscope appropriately in order to assist the operator in transporting an object (plastic cylinder, 2 cm in diameter) between three pegs located at three different sides of a cardboard box (18 × 15 × 9 cm) inside the training box (Fig. 1). It is essential for the assistant to understand the optical properties of the laparoscope and how to manipulate the scope to keep the object and instruments in the center of the field of view. The collaborative suturing task required the assistant to remove an obstacle (rubber band) placed on top of the surgical site so that the operator could perform a successful intracorporeal suture (Fig. 1). During the task, the assistant must control the laparoscope to view the suture site, the suture needles, and the movements of a pair of needle drivers in the hands of the operator.

The two tasks included in this study address the required individual and team skills for performing a laparoscopic surgery. At the individual level, both participants must scan the surgical site, control the laparoscopy equipment, and develop eye-hand and bimanual coordination skills. At the team level, the tasks required the two team members to develop the ability to coordinate their movements and to communicate clearly with each other [25].

### Procedure

Each 2-person team was required to perform the two tasks under three different acoustic conditions. The order of the acoustic conditions were randomized to counterbalance possible learning effect in the practice. Each team was allowed a 10-minute break between acoustic conditions to minimize individual biases. Each participant in the team was required to wear headsets that delivered the acoustic condition at 45 dB (decibel) and canceled out ambient sounds while the participant performed the tasks.

In the pleasant and smooth music condition, we played the To Alice (Piano Sole) composed by Beethoven. The selection of this piece was straightforward. A quick survey of thirteen healthcare staff in the simulation center on their feelings after listening to this piece of masterpiece, three words were most frequently mentioned: “Pleasant” (100%), “Delightful” (92%), and “Romantic” (85%). To Alice was listed as the most popular piece of classic music in QQ Music Website (Tencent Holding Ltd., Shenzhen, China) with thousands of comments indicating that listening to it induced pleasant feeling .

In the noisy environment, we played a piece of soundtrack at 45 dB recorded from the operating room. The soundtrack was downloaded from QQ Music website (https://y.qq.com/). Aloud but imperceptible human conversation, phone rings, cart moving, sound from tearing up a plastic package and beeping sounds from vital signs monitors can be heard in this piece of acoustic soundtrack. In the neutral group, we turned off all acoustic records. Participants were required to wear a headset (Harman, JDN2-W09, Huawei Technologies Co Ltd., Shenzhen, China) at all times, which provided a quiet environment in the neutral condition.

### Measures

Task performance was recorded via a digital camera connected to the laparoscopic tower. Video analysis was later performed by one experienced surgeon, who was unaware of the study purposes. This surgeon examined the videos, labeled the starting and ending points of subtasks in each video, and counted the errors performed by the residents in each team. These measures were then used to generate a summative task score and a total score for the performance of each team.

Table 1 lists the times for completing each task, subtask, and the number of errors for each of the two tasks, and provides a detailed description of each of the measures. The times of each task were calculated by subtracting the completion time with the start time of the task. A penalty (three seconds) was added to the task time for each error observed.

The recorded errors were further divided into individual and team errors. For example, dropping an object and dropping the needle when adjusting position were considered individual errors, whereas misalignment of laparoscope, collision of instruments, and miscommunications were considered as team errors. A secure knot received a zero penalty, a slipping knot received a 10-second penalty, and a knot received a 20-second penalty, as based on Derossis’ scoring system for suture quality [28].

A total score for an operation was obtained by adding the penalty times to the time taken to complete the task. To adjust the total score of each task so that it was comparable to the other task, we normalized the total score of each task to the maximum value recorded during the task, using the equations below:

$$Normalized\ object\ transportation\ score=\left( Maximum- total\ score\ of\ trial\right)/ Maximum\times 100$$

$$Normalized\ suturing\ score=\left( Maximum- total\ score\ of\ teach\ trial\right)/ Maximum\times 100$$

$$Normalizedtaskscore=\left( Normalized\ object\ transportation\ score+ Normalized\ suturing\ score\right)/2$$

The total team score was averaged by taking two team members’ transportation and suturing scores.

$$Team\ score=\left( Normalized\ individual\ score\ 1+ Normalized\ individual\ score\ 2\right)/ 2$$

The more accurately and quickly a task was completed by each team member, the higher the individual and team scores were.

#### Post-test assessment on team quality

At the end of each operation, the participants of each team were required to evaluate their team performance in terms of quality of interpersonal communication and cooperation. Each team member used a 10-point scale (1 the worst and 10 the best) to answer eight questions. A mean quality score was determined from the sum of the two self-rated scores from each of the two team members.

### Statistical analysis

We eventually recruited 57 teams to test our hypothesis with SPSS Statistics 22.0 (IBM Corporate, Armonk, New York, USA). Variables such as task times and penalties were compared between the three acoustic conditions by one-way within-subject ANOVA. Post-hoc pairwise comparisons were performed by the Bonferroni method. Pearson’s *r* (correlation coefficient) was calculated to examine the correlation between the team experience score and the surgical performance score. Data were reported as means ± standard deviations. *p* < 0.05 was considered statistically significant.

## Results

### Demographics

This study enrolled 114 surgeons. The demographics of the participants are listed in Table 2. Most of the participants were in the early stages of laparoscopic practice, and reported a mean duration of training in laparoscopy of 2.4 years. The score of surgical experience were determined by asking each participant to report the number of the basic and advanced laparoscopic procedures that he or she had performed by the time of the study [25]. The POMS score of the participants was 103.8 ± 3.6, indicating that the participants were calm before the operations. The PANAS-SF scored 27.1 ± 7.2 and 17.0 ± 7.0 respectively, which confirmed that the participants felt positive before the operations. Since each participant was required to become a member of a 2-person team and undergo all three acoustic conditions in a randomized order according to the counterbalanced measures design, we did not divide them further into different experimental groups and compare their demographic characteristics.

**Table 2:** Demographics of participants before they entered the study

Number of resident	114
Dyad Team	57
Age	34.3 ± 4.8
Male:Female	88:26
Years in Lap Surgical Training	2.4 ± 1.1
Surgical Experience Score	57.2 ± 19.8
POMS	103.8 ± 3.6
PANAS Positive	27.1 ± 7.2
PANAS Negative	17.0 ± 7.0

### Impact of acoustic conditions on performance

One way within-subject ANOVA revealed significant differences on the acoustic conditions from all team performance variables, except for Penalty Operator OT and Penalty Operator OT (Table 3). The time to perform a task under the pleasant and smooth music (65.9 ± 17.8 s) was shorter than the time to perform tasks in a neutral (74.9 ± 19.9 s) and a noisy environment (90.4 ± 24.1 s; *p* < 0.001). A *post-hoc* pairwise comparison found significant differences between the music and noisy environments. Compared with the neutral environment, the noisy environment led to significantly more negative effects than the music environment which led to a positive impact on performance.

**Table 3:** Statistical outputs on time and error measures compared between 3 different acoustic conditions. Surgical performance under three auditory conditions

		Music	Neutral	Noisy	F	***p*** Value	***p*** Value (post hoc)		
						Auditory Condition	Music vs Noisy	Music vs Neutral	Noisy vs Neutral
Object Transportation (OT)	Total OT time (s)	65.9 ± 17.8	74.9 ± 19.9	90.4 ± 24.1	20.3	< 0.001	< 0.001	0.066	< 0.001
	Subtask A Time (s)	34.2 ± 8.8	39.6 ± 12.2	48.2 ± 14.7	19.3	< 0.001	< 0.001	0.054	< 0.001
	Subtask B Time (s)	17.9 ± 5.4	20.0 ± 6.0	25.3 ± 9.3	16.4	< 0.001	< 0.001	0.342	< 0.001
	Subtask C Time (s)	7.8 ± 3.3	8.5 ± 3.1	9.7 ± 3.4	5.4	0.006	0.005	0.784	0.113
	Penalty Operator OT	6.3 ± 0.8	6.9 ± 2.6	6.6 ± 2.1	1.1	0.329	0.935	0.411	0.877
	Penalty Team OT	59.2 ± 23.2	61.2 ± 24.3	68.4 ± 27.9	2.1	0.126	0.161	0.914	0.382
	Total OT Score	131.4 ± 33.7	142.9 ± 36.3	165.4 ± 43.7	11.7	< 0.001	< 0.001	0.328	0.006
	Normalized OT Score	66.3 ± 8.6	63.4 ± 9.3	57.6 ± 11.2	11.7	< 0.001	< 0.001	0.324	0.006
Collaborative Suturing (CS)	Total Suture Time (s)	165.1 ± 41.5	210.4 ± 69.3	262.6 ± 92.7	26.873	< 0.001	< 0.001	0.002	< 0.001
	Suture Preparation Time (s)	26.5 ± 9.8	40.1 ± 24.4	62.7 ± 45.3	20.826	< 0.001	< 0.001	0.052	< 0.001
	Suture Needle time (s)	23.5 ± 10.7	30.7 ± 18.2	34.5 ± 33.3	3.431	0.035	0.032	0.277	0.73
	1st Knot Time (s)	55.9 ± 25.8	69.3 ± 34.5	87.4 ± 45.9	10.810	< 0.001	< 0.001	0.148	0.026
	2nd Knot Time (s)	26.7 ± 12.4	35.2 ± 17.8	38.9 ± 21.2	7.359	0.001	0.001	0.029	0.784
	Cutting Time (s)	24.3 ± 9.7	24.7 ± 11.3	28.9 ± 12.1	3.107	0.047	0.046	0.976	0.127
	Penalty Operator CS	9.7 ± 7.1	14.6 ± 9.1	21.2 ± 12.4	3.104	< 0.001	< 0.001	0.025	0.001
	Penalty Team CS	41.1 ± 13.7	44.3 ± 14.7	48.3 ± 17.3	20.008	0.047	0.042	0.83	0.495
	Penalty Total CS	53.7 ± 17.5	62.1 ± 20.1	73.3 ± 26.4	11.722	< 0.001	< 0.001	0.128	0.018
	Total CS Score	218.8 ± 54.8	272.4 ± 84.14	335.9 ± 113.6	25.540	< 0.001	< 0.001	0.004	< 0.001
	Normalized Suture Score	78.6 ± 5.4	73.4 ± 8.2	67.2 ± 11.1	25.540	< 0.001	< 0.001	0.004	< 0.001

### Surgical experience vs. acoustic distraction

**Fig. 2:**
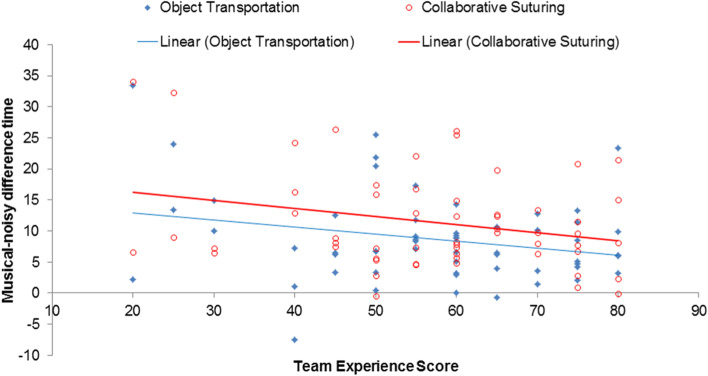
Scatter plots displaying the correlations between team experience scores and music-noisy difference time for the object transportation (blue) and collaborative suturing (red) tasks

We noticed different teams showed different degrees of resistance to an acoustic condition. We subtracted task times between a music and noisy environment to obtain a *music-noisy difference time* to describe the impact of the noisy environment on team performance as opposed to pleasant and smooth music. A large time gap between the music and noisy environments indicated that the impact on team performance was large. The correlation between team-experience scores and the music-noisy difference time for object transportation was *r* = − 0.246 (*p* = 0.046) and collaborative suturing *r* = − 0.248 (*p* = 0.044) tasks. As shown in Fig. 2, the team with a higher score for surgical experience showed a smaller difference between the music and noisy environments.

### Post-test assessment

At the end of each operation, team members were required to evaluate their team performance. On average, they reported 8.6 ± 0.9 points on a 10-point scale, a good sign for team collaboration by self-reported assessment.

## Discussion

The findings of this study support our hypothesis. Laparoscopic surgical teams working in the music environment perform significantly better than the same teams working in the noisy environment. In particular, the teams took significantly less time to complete the collaborative suturing tasks with fewer time penalties with pleasant and smooth music than the noisy environment in the background (Table 3). The exact reason behind the positive effect of pleasant and smooth music to surgeons performance is not fully known. It may induce delighted positive mood in surgeons on a surgical team. In contrast, exposure to a noisy environment led to a prolonged task time and increased the number of errors committed by both individual team members in a team. A noisy environment added cognitive loads to all team members, reducing their mental resources for processing information during the surgery, with resultant delays for decision-making and reductions in controlled movements [14, 18]. The opposite effect was observed when operators worked in an environment with their preferred music playing. In this study, unfortunately, we did not assess the combined effects of music and noisy environments. In future research, we will examine if music will provide a benefit to operators working in a noisy environment.

The exciting finding from this study is that collaborative teams seemed to show increased resistance to poor acoustic conditions. Surgical teams consisting of experienced surgeons displayed more resistance to change from a music to a noisy environment than teams consisting of inexperienced surgeons. This finding suggests that collaborative teams can accommodate the negative impact of acoustic distraction and maintain their performance in those difficult and demanding environments such as in disasters and war conflicts. Our interpretation of the results regarding collaborative teams is consistent with previous studies [29–31]. Large surgical tasks become automatic in surgeons who have become skillful. The cognitive resources of skillful team members allow them to manage environmental distractions [30]. Therefore experienced team members have increased ability in dealing with extra environmental feedback while performing a task.

The negative linear relationship between team experience and impact of acoustic condition creates an opportunity for us to assess the skills of a surgical team by examining its resistance to environmental noise. This could inspire a new approach additional to the array of other assessment instruments currently used for assessing surgical team performance. Further validation is needed before we can comfortably introduce this approach for assessing team performance.

Results from our study suggest that playing pleasant and smooth music in the operating room enhances surgical team performance during laparoscopic procedure. However, this result may not be able to generalize to other surgical specialties as collaboration pattern between surgeons can be different.

There are other limitations to the current study. First, the performance videos were analyzed by one single surgeon; we may not rule off the personal bias. However, this reviewer did not know the purpose of the study and was blind to the experimental condition. Second, we were unable to report on the effect of different types of music stimulation provided to each of the two team members. We did not assess the mood value before and after the training. In future research, we will make these data available for in-depth analysis. Third, the experimental setup in the study did not completely reflect the actual operating conditions. The testing tasks were relatively easy to perform. Additionally, an actual surgical procedure consists of a laparoscopic team that includes nurses, anesthesiologists, and technicians. Future research should consider to enroll entire surgical teams that perform laparoscopy as the study participants.

## Conclusions

Results from our study indicated that laparoscopic teams performed better in a pleasant and smooth music than in a noisy environment in the simulated settings. Surgeons with a higher experience score were less affected by a noisy environment which suggest their resistance to the unpleasant acoustic environment. Team resistance to acoustic distraction may open an opportunity for assess team collaboration quality to fulfill our long-term goal of improving surgical team performance and patient safety.

## Data Availability

The datasets used and/or analysed during the current study available from the corresponding author on reasonable request. The video recordings, which contain identifiable images of the study subjects, are not to be shared for privacy protection concerns.
